# Captive Rearing of Longfin Smelt *Spirinchus thaleichthys*: First Attempt of Weaning Cultured Juveniles to Dry Feed

**DOI:** 10.3390/ani12121478

**Published:** 2022-06-07

**Authors:** William Mulvaney, Md Moshiur Rahman, Levi S. Lewis, Jiayi Cheng, Tien-Chieh Hung

**Affiliations:** 1 Fish Conservation and Culture Laboratory, Department of Biological and Agricultural Engineering, University of California, 17501 Byron Hwy, Byron, Davis, CA 94514, USA; will.j.mulvaney@gmail.com (W.M.); momrahman@ucdavis.edu (M.M.R.); jicheng@ucdavis.edu (J.C.); 2 Otolith Geochemistry and Fish Ecology Laboratory, Department of Wildlife, Fish and Conservation Biology, University of California, 1088 Academic Surge, Davis, CA 95616, USA; lslewis@ucdavis.edu

**Keywords:** Osmeridae, ex situ conservation, diet, fatty acids, anadromous fish

## Abstract

**Simple Summary:**

Longfin smelt *Spirinchus thaleichthys* is an imperiled estuarine species in California, USA. A captive culture program is currently being developed; however, prior to this study, longfin smelt have only fed on live prey in the hatchery. Here, we report on our first successful attempt to wean cultured juveniles onto a dry commercial pellet feed. A subset of F1 fish was switched to a mixed diet of reduced *Artemia* and dry feed for 62 days, with growth, survival, and body condition compared between feeding treatments. Our results highlight that juvenile longfin smelt can utilize dry feeds while maintaining a healthy body condition.

**Abstract:**

The rapid decline of longfin smelt *Spirinchus thaleichthys*, a threatened euryhaline forage fish in California, is a serious concern for scientists and resource managers. To recover and conserve this species, a captive culture program was initiated, focusing on the collection, captive rearing and breeding of wild broodstock, and the rearing of their offspring. Although progress has been made in the collection of broodstock and the production and culturing of larvae, no studies have evaluated the rearing of juvenile life stages in captivity. The present study examines methodological considerations for culturing F1 juvenile longfin smelt, specifically, the first efforts toward weaning juveniles to a dry commercial pellet feed. Cultured juvenile longfin smelt were fed live *Artemia* only or co-fed *Artemia* and dry feed for 62 days, and the effects of feed type on juvenile survival, growth, body condition, and fatty acid profiles were examined. No significant differences were observed between feeding treatments, despite an 80% reduction in *Artemia* in the co-feeding treatment. Furthermore, examination of fish stomach contents at the end of the trial confirmed the transition to dry feed. This is the first study to indicate successful feeding by longfin smelt on dry commercial pellets, and suggests that juvenile longfin smelt can be fully weaned onto dry feeds. Results of this study are critical for closing the lifecycle of longfin smelt in captivity and developing a successful conservation culture program for this imperiled species.

## 1. Introduction

The rapid decline of longfin smelt *Spirinchus thaleichthys*, a threatened euryhaline forage fish in California, is a serious concern for scientists and resource managers [1]. As one strategy to help recover and conserve this species, the Fish Conservation and Culture Laboratory (FCCL) at the University of California, Davis, has been working to develop a captive culture program for longfin smelt [2,3]. To maximize the success of these efforts, studies examining longfin smelt under captive conditions are needed. Although recent studies have helped identify suitable rearing conditions for newly hatched larvae [4,5], no published studies have examined the collection of broodstock, spawning of broodstock, or the rearing of juvenile and adult life stages in captivity. The incorporation of dry feeds into the diets of cultured populations is a critical step in closing the lifecycle and establishing a robust captive culture program [6]; however, previous attempts at weaning longfin smelt to dry feeds at any life stage have been unsuccessful.

Here, we examined the responses of cultured F1 juvenile longfin smelt to commercial dry pellet feed. Fish received either *Artemia* alone or a mixed diet of reduced *Artemia* plus dry pellets, thus allowing us to explore the effects of each feed type on juvenile survival, growth, body condition, and fatty acid profiles. Results of this study will provide key information to support the development of a successful longfin smelt culture program that can be used to conserve this imperiled species.

## 2. Materials and Methods

### 2.1. Broodstock Collection, Spawning, and Larviculture

During the 2019–2020 spawning season (November 2019–March 2020), 264 live wild mature (fork length = 90.1 ± 11.2 mm) longfin smelt were collected and transported from the San Francisco Estuary to the FCCL (Table S1). All fish were quarantined and given a 3-day prophylactic antibiotic treatment (Pennox 343, Animal Health International, Ceres, CA, USA) in standing water with aeration (20 NTU, 10 ppt, and 12 °C). After quarantine, fish were measured, tagged, and consolidated in 400-L broodstock holding tanks. The stocking density for each tank was set to be 50 adult fish per tank. The tanks received 500 mL d^−1^ live adult brine shrimp *Artemia franciscana* (Artemia International, Fairview, TX, USA) as feed. Ripe individuals were strip-spawned following established protocols [4] to produce multiple crosses of cultured F1 longfin smelt. The eggs were fertilized and incubated in pre-treated source water from the California Aqueduct (Contra Costa County, CA, USA) with a salinity of 0.4 ppt and temperature of 12 °C. Larvae were hatched after a 16-day incubation period and held in 2 ppt at 12 °C and fed rotifers *Brachionus plicatilis* (L-type) with a size of about 210 µm (Reed Mariculture, Pasadena, CA, USA) and newly hatched, unenriched *Artemia* nauplii. As fish grew, surviving larvae and juveniles with the same age from multiple crosses were pooled together.

### 2.2. Experimental Design

At 160 days post hatch (dph), 228 juvenile F1 longfin smelt (fork length = 34.3 ± 3.6 mm) were randomly assigned to nine 400-L juvenile rearing tanks (38 fish per tank). Tanks were arranged in groups of 3, with each group plumbed to a separate recirculating aquaculture system (2 systems; 3 tanks per system). Both systems were controlled to have the same environmental conditions to match conditions commonly experienced by juvenile longfin smelt (temperature = 12 °C; salinity = 5 ppt). Juveniles were held for a 7-day acclimation period, during which all fish were fed newly hatched, unenriched *Artemia* nauplii.

After the acclimation period, each tank was randomly assigned to one of two feeding treatments. Treatment 1 (*Artemia*, ART) continued to receive live newly hatched, unenriched *Artemia* nauplii at 500 mL per day (~2250 nauplii mL^−1^), provided as 5 × 100 mL feedings per day. Treatment 2 (dry feed + *Artemia*, DFA) consisted of a combined diet of 1 g per day dry commercial feed (Biovita Starter mash crumble, Bio-Oregon, Longview, WA; provided as 0.25 g feedings 4 times per day) and 100 mL *Artemia* provided only once at the end of each day (Table 1). Thus, Treatment 2 reflected an 80% reduction in *Artemia* relative to the ART treatment, with dry feed providing supplemental nutrition. All live *Artemia* were grown on site, and all the fish tanks were siphoned 3 times a week to remove feces and uneaten food and checked daily for any mortalities. The study was carried out for 62 days, after which 10 fish were sampled from each tank for further analysis (see Section 2.3).

After the 62-day feeding trial was completed and subsampled, the remaining fish from each treatment were consolidated into separate 400-L tanks. The ART group continued to be fed *Artemia* only while the DFA group was transitioned to 100% dry feed (DF, without *Artemia*). Survival in both tanks was monitored for an additional 66 days (from 229–295 dph), with survival in the ART and DF treatments being assessed at the end of the trial. All fish were then further consolidated into a single tank and fed dry feed only, and the feeding study was completed.

### 2.3. Size, Condition, and Tissue Analysis

At the end of the 62-day feeding trial, 30 individuals from each treatment (10 fish per tank) were sampled, euthanized with 500 mg L^−1^ buffered tricaine methanesulfonate (MS-222, IACUC Protocol #21353), and their fork lengths (FL, in cm) and body weights (BW, in g) were measured using a measuring board and electronic balance (PW124, Adam Equipment, Inc., Oxford, CT, USA), respectively. Fulton’s condition factor (K) was then calculated from BW and FL using the formula of Froese [7]. Muscle tissue from these was then collected from several fish from each tank and pooled (to a total of 3 g wet mass) for fatty acid and dry matter analyses. The profiling of different fatty acids was done at the UC Davis Fiehn lab (https://metabolomics.ucdavis.edu/; accessed on 27 May 2021) using ultra-high pressure liquid chromatography (UPLC) following the methods of Matyash et al. [8]. Each component of fatty acid was estimated as a percentage of the total lipid content.

### 2.4. Statistical Analysis

All analyses and plotting were done using R version 4.0.2 [9]. The assumptions of normality and homogeneity were checked with the Shapiro–Wilk test and Levene’s test, respectively, using the ‘onewaytests’ package. Variations between feed treatments in survival, growth, condition factor, and fatty acids were examined with *t*-tests. Significance was defined as *p* < 0.05. 

## 3. Results and Remarks

### 3.1. Weaning Success, Survival, Growth, and Condition

**Figure 1:**
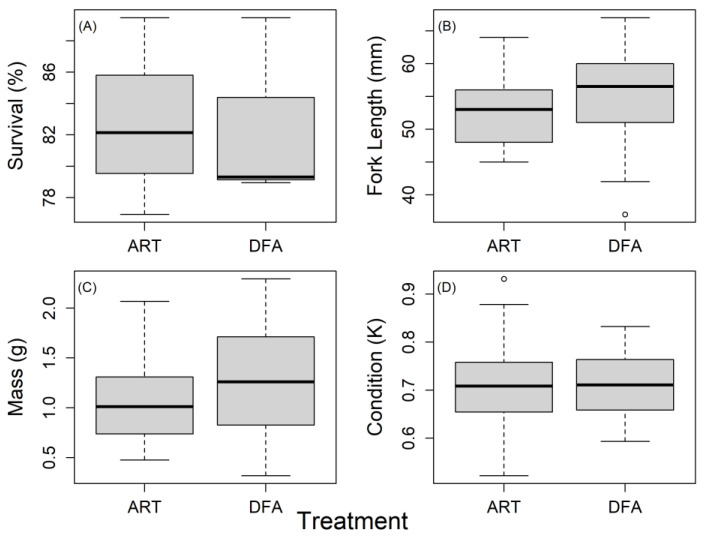
(**A**) Survival (%), (**B**) fork length (mm), (**C**) mass (g), and (**D**) condition of fish in each feeding treatment (*n* = 30 per treatment) at the end of the 62-day trial. ART—*Artemia* only group; DFA—dry feed plus 80% reduction in *Artemia*. IQR = box (25–75th percentiles), and ‘whiskers’ are the 1.5 IQR or min/max if all values < 1.5 IQR.

Dry feed was regularly observed in the guts of the juvenile longfin smelt collected at the end of the 62-day feeding trial, confirming that fish were consuming pellet feed. Survival rates were 79.82 ± 4.88% and 82.58 ± 3.45% for DFA and ART, respectively, and did not differ significantly among treatments (*t*-test; *p* = 0.96, Figure 1A). The survival of ART and DF groups at 66 days after the trial was 96.6% and 100.0%, respectively. Similarly, no differences were observed between treatments in fork length (*t*-test, *p* = 0.17, Figure 1B), weight (*t*-test; *p* = 0.21, Figure 1C), nor K-value (*t*-test; *p* = 0.82, Figure 1D). Thus, juvenile longfin smelt were successfully transitioned onto dry feed, with survival, growth, and body condition of these fish similar to those fed a live-feed only diet.

### 3.2. Fatty Acid Profile of Artemia and Dry Pellet Feeds

**Figure 2:**
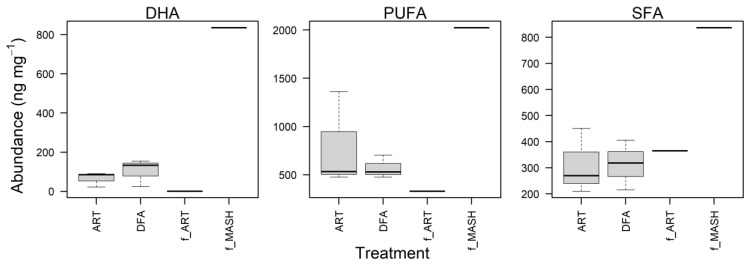
Differences in DHA, PUFA, and SFA levels of muscle tissue from juvenile longfin smelt in the ART and DFA feeding groups and in the feed used (f_ART—live *Artemia* and f_MASH—dry feed). IQR = box (25–75th percentiles), and ‘whiskers’ are the 1.5 IQR or min/max if all values < 1.5 IQR.

Dry feed contained higher amounts of saturated fatty acids (SFAs) and polyunsaturated fatty acids (PUFAs) than live *Artemia* even though live *Artemia* contained a relatively higher percentage of saturated fatty acids in its total fatty acids (Figure 2; Table S2). The ratios of n3:n6 were higher in newly hatched *Artemia* (3.32, while it was 0.16 in dry feed), whereas EPA:ARA and DHA:EPA were higher in dry feed (34.05 and 1.41, respectively, while they were 1.76 and 0.05, respectively, in *Artemia*). Essential fatty acids are crucial for the survival, development, and physiology of fish larvae [10,11,12], and high levels of DHA are often related to growth and body condition [13,14,15]. DHA level in the dry feed used in this study was higher than in the newly hatched *Artemia* (21.52% and 0.08%, respectively). Therefore, including of dry feed may be a good strategy to better meet the nutritional requirements of the fish [16,17].

### 3.3. Fatty Acid Profile of Juvenile Muscle Tissues

**Figure 3:**
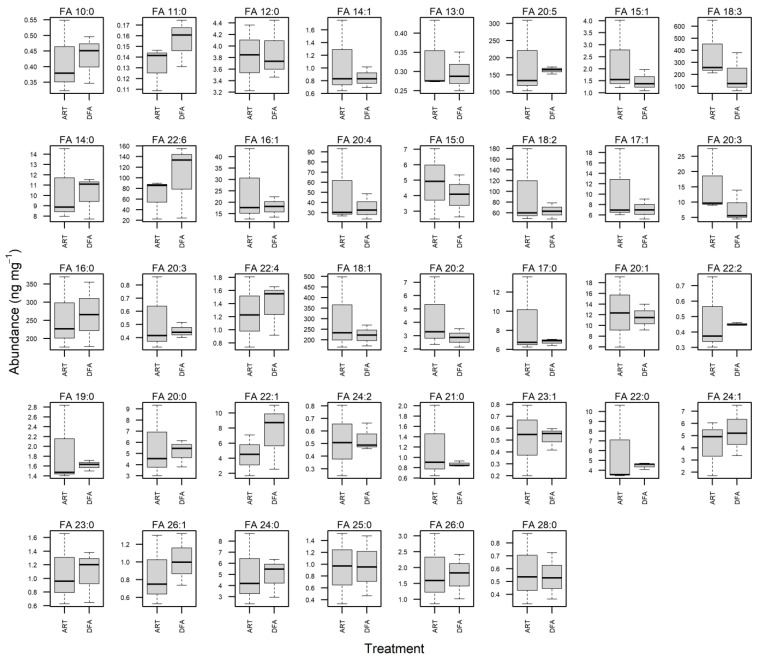
Differences in fatty acid levels of muscle tissue from juvenile longfin smelt in the ART and DFA feeding groups. IQR = box (25–75th percentiles), and ‘whiskers’ are the 1.5 IQR or min/max if all values < 1.5 IQR.

Dry matter mass (% wet weight) of muscle tissue from juvenile longfin smelt did not appear to differ between feeding treatments (*t*-test; *p* = 0.73). Regarding the essential fatty acids, fish in the DFA group had slightly higher levels of DHA than the ART group (Figure 2), though insignificant (Figure 3; Table S3). In aggregate, these results indicate that juvenile longfin smelt weaned on dry feed were able to meet their nutritional requirements and maintained similar tissue and fatty acid compositions when compared to the fish fed live prey alone. Given that longfin smelt exhibit a migratory life history, it remains crucial that we continue exploring their optimal rearing conditions and nutritional requirements as they experience ontogenetic physiological changes throughout their development [18,19,20].

## Figures and Tables

**Table 1:** Experimental design of the 62-day feeding experiment.

Feed Treatment	N_fish_	Feed Quantity	Frequency
*1. Artemia*^a^ (ART)	114	500 mL	100 mL; 5 × daily
2. Dry feed ^b^ + *Artemia (DFA)*	114	1.0 g (dry feed) + 100 mL (*Artemia*)	0.25 g; 4 × daily 100 mL 1 × daily

^a^ Live adult *Artemia franciscana*; ^b^ a commercial dry feed (Biovita Starter mash crumble).

## Data Availability

Data are available upon request to the corresponding author.

## Supplementary Materials

The following supporting information can be downloaded at: https://www.mdpi.com/article/10.3390/ani12121478/s1, Table S1: Summary of wild longfin smelt broodstock collected during the 2019-2020 spawning season. Fork length is presented as means ± SD. Surveys are FCCL: Fish Conservation and Culture Laboratory; OGFL: UC Davis Otolith Geochemistry & Fish Ecology Laboratory (OGFL); and USFWS: U.S. Fish and Wildlife Service (USFWS) Chipps Island Trawl; Table S2: Fatty acids profile from the total lipid contents (%) of different types of feed; Table S3: Fatty acids profile from the total lipid contents (%) of juvenile longfin smelt reared with different types of feed.
