# Supplementary material for: Captive Rearing of Longfin Smelt Spirinchus thaleichthys: First Attempt of Weaning Cultured Juveniles to Dry Feed
Source: Animals (Basel). 2022 Jun 7;12(12):1478. doi: 10.3390/ani12121478 (PMC9219441; doi:10.3390/ani12121478)
Supplement: Supplementary file 1 [file animals-12-01478-s001.zip › animals-1738961-supplementary.pdf]

animals

MDPI

Supplementary Materials

Captive Rearing of Longfin Smelt *Spirinchus thaleichthys*: First Attempt of Weaning Cultured Juveniles to Dry Feed

William Mulvaney [1], Md Moshiur Rahman [1], Levi S. Lewis [2], Jiayi Cheng [1] and Tien-Chieh Hung [1,*]

[1] Fish Conservation and Culture Laboratory, Department of Biological and Agricultural Engineering, University of California, 17501 Byron Hwy, Byron, Davis, CA 94514, USA; will.j.mulvaney@gmail.com (W.M.); momrahman@ucdavis.edu (M.M.R.); jicheng@ucdavis.edu (J.C.)
[2] Otolith Geochemistry and Fish Ecology Laboratory, Department of Wildlife, Fish and Conservation Biology, University of California, 1088 Academic Surge, Davis, CA 95616, USA; lslewis@ucdavis.edu
* Correspondence: thung@ucdavis.edu

Supplemental Tables

Table S1. Summary of wild longfin smelt broodstock collected during the 2019-2020 spawning season. Fork length is presented as means ± SD. Surveys are FCCL: Fish Conservation and Culture Laboratory; OGFL: UC Davis Otolith Geochemistry & Fish Ecology Laboratory (OGFL); and USFWS: U.S. Fish and Wildlife Service (USFWS) Chipps Island Trawl.

Survey	Number of Fish	Collection Gear	Sampling Duration	Fork Length (mm)
FCCL	14	lampara net	Nov 2019–Dec 2019	88.2 ± 17.1
OGFL	201	benthic otter trawl	Dec 2019–Jan 2020	89.3 ± 11.1
USFWS	49	midwater trawl	Dec 2019–Mar 2020	93.9 ± 8.7

Table S2. Fatty acids profile from the total lipid contents (%) of different types of feed.

Common name	Structure	Fatty Acids Content (% of Total Lipid) in Different Feeds	
		Artemia	Dry Feed
Capric acid (CA)	10:0	0.03	0.05
Undecylic acid (UA)	11:0	0.01	0.01
Lauric acid (LA)	12:0	0.39	0.14
Tridecylic acid (TA)	13:0	0.06	0.02
Myristic acid (MRA)	14:0	1.44	2.11
Pentadecylic acid (PDA)	15:0	0.84	0.49
Palmitic acid (PA)	16:0	30.03	16.04
Margaric acid (MGA)	17:0	1.04	0.64
Octylcyclopropyl-octanoic acid (OOA)	19:0	0.15	0.17
Arachidic acid (ARA)	20:0	0.84	0.45
Methylhenicosanoic acid (MHA)	21:0	0.06	0.05
Behenic acid (BA)	22:0	0.48	0.30
Trimethyltricosanoic acid (TTA)	23:0	0.16	0.11
Lignoceric acid (LCA)	24:0	0.75	0.55
Pentacosanoic acid (PCA)	25:0	0.21	0.06
Cerotic acid (CTA)	26:0	0.33	0.24
Montanic acid (MA)	28:0	0.09	0.07
Physeteric acid (PSA)	14:1	0.25	0.05
Pentadec-8-enoic acid (PEA)	15:1	0.20	0.08

Palmitoleic acid (PMA)	16:1	2.65	3.67
Heptadec-enoic acid (HEA)	17:1	0.83	0.73
Oleic acid (OA)	18:1	24.66	14.02
Eicosenoic acid (ESA)	20:1	0.71	3.28
Erucic acid (ECA)	22:1	0.39	3.13
Tricosenoic acid (TSA)	23:1	0.01	0.06
Nervonic acid (NVA)	24:1	0.09	1.26
Hexacos-enoic acid (HXA)	26:1	0.04	0.20
Linoleic acid (LLA)	18:2	7.02	9.75
Linolenic acid (LNA)	18:3	23.33	1.52
Eicosadienoic acid (EDA)	20:2	0.21	0.33
Homo-gamma-linolenic acid (HGLA)	20:3	5.77	9.96
Eicosatrienoic acid (ETA)	20:3	0.05	0.14
Arachidonic acid (ADA)	20:4	0.44	3.01
Eicosapentaenoic acid (EPA)	20:5	1.47	15.17
Docosadienoic acid (DSA)	22:2	0.04	0.07
Tetraenoic acid (TEA)	22:4	0.03	0.23
Docosahexaenoic acid (DHA)	22:6	0.08	21.47
Bronsted acid (BSA)	24:2	0.01	0.05
Saturated fatty acids (SFAs)	-	36.89	21.50
Monounsaturated fatty acids (MUFAs)	-	29.85	26.50
Polyunsaturated fatty acids (PUFAs)	-	33.26	52.00
Total fatty acids (TFAs)	-	100.00	100.00
n3:n6	-	3.32	0.16
EPA:ARA	-	1.76	34.05
DHA:EPA	-	0.05	1.41

Analysis and comparison were not made because of only one sample per feed was taken during fatty acid profiling.

Table S3. Fatty acids profile from the total lipid contents (%) of juvenile longfin smelt reared with different types of feed.

Common Name	Structure	Fatty Acids Content (% of Total lipid) in Different Treatments		t	*p*
		Artemia	Dry feed + *Artemia*		
Capric acid (CA)	10:0	0.03±0.00^{a}	0.04±0.00^{a}	-1.23	0.29
Undecylic acid (UA)	11:0	0.01±0.00^{a}	0.01±0.00^{a}	-1.06	0.35
Lauric acid (LA)	12:0	0.30±0.07^{a}	0.34±0.02^{a}	-0.50	0.64
Tridecylic acid (TA)	13:0	0.02±0.00^{a}	0.03±0.00^{a}	-0.27	0.80
Myristic acid (MRA)	14:0	0.78±0.10^{a}	0.90±0.15^{a}	-0.65	0.55
Pentadecylic acid (PDA)	15:0	0.36±0.09^{a}	0.36±0.09^{a}	-0.03	0.97
Palmitic acid (PA)	16:0	19.01±2.67^{a}	23.35±4.47^{a}	-0.83	0.45
Margaric acid (MGA)	17:0	0.64±0.05^{a}	0.60±0.05^{a}	0.57	0.60
Octylcyclopropyl-octanoic acid (OOA)	19:0	0.14±0.01^{a}	0.14±0.01^{a}	-0.23	0.83
Arachidic acid (ARA)	20:0	0.39±0.05^{a}	0.45±0.07^{a}	-0.67	0.53
Methylhenicosanoic acid (MHA)	21:0	0.08±0.01^{a}	0.08±0.00^{a}	0.67	0.54
Behenic acid (BA)	22:0	0.39±0.03^{a}	0.39±0.01^{a}	-0.04	0.97
Trimethyltricosanoic acid (TTA)	23:0	0.08±0.01^{a}	0.10±0.02^{a}	-0.67	0.54
Lignoceric acid (LCA)	24:0	0.35±0.06^{a}	0.44±0.11^{a}	-0.71	0.51
Pentacosanoic acid (PCA)	25:0	0.07±0.02^{a}	0.09±0.03^{a}	-0.56	0.61
Cerotic acid (CTA)	26:0	0.13±0.03^{a}	0.16±0.05^{a}	-0.57	0.60
Montanic acid (MA)	28:0	0.04±0.01^{a}	0.05±0.01^{a}	-0.43	0.69

Physeteric acid (PSA)	14:1	0.08±0.01[a]	0.08±0.01[a]	-0.01	0.99
Pentadec-8-enoic acid (PEA)	15:1	0.15±0.02[a]	0.13±0.02[a]	0.84	0.45
Palmitoleic acid (PMA)	16:1	1.64±0.14[a]	1.55±0.15[a]	0.43	0.69
Heptadec-enoic acid (HEA)	17:1	0.71±0.04[a]	0.61±0.07[a]	1.19	0.30
Oleic acid (OA)	18:1	20.51±1.51[a]	19.11±1.55[a]	0.65	0.55
Eicosenoic acid (ESA)	20:1	0.88±0.17[a]	1.01±0.13[a]	-0.61	0.58
Erucic acid (ECA)	22:1	0.36±0.17[a]	0.66±0.23[a]	-1.08	0.34
Tricosenoic acid (TSA)	23:1	0.04±0.01[a]	0.05±0.01[a]	-0.88	0.43
Nervonic acid (NVA)	24:1	0.31±0.09[a]	0.47±0.10[a]	-1.20	0.30
Hexacos-enoic acid (HXA)	26:1	0.06±0.01[a]	0.09±0.01[a]	-1.73	0.16
Linoleic acid (LLA)	18:2	6.24±0.68[a]	5.47±0.48[a]	0.92	0.41
Linolenic acid (LNA)	18:3	25.20±2.17[a]	15.91±7.69[a]	1.16	0.31
Eicosadienoic acid (EDA)	20:2	0.29±0.02[a]	0.25±0.02[a]	1.56	0.19
Homo-gamma-linolenic acid (HGLA)	20:3	1.01±0.08[a]	0.68±0.23[a]	1.40	0.23
Eicosatrienoic acid (ETA)	20:3	0.04±0.00[a]	0.04±0.01[a]	-0.37	0.73
Arachidonic acid (ADA)	20:4	3.26±0.38[a]	3.01±0.50[a]	0.40	0.71
Eicosapentaenoic acid (EPA)	20:5	12.40±0.63[a]	14.40±1.41[a]	-1.29	0.27
Docosadienoic acid (DSA)	22:2	0.03±0.00[a]	0.04±0.00[a]	-1.72	0.16
Tetraenoic acid (TEA)	22:4	0.09±0.01[a]	0.12±0.02[a]	-1.07	0.34
Docosahexaenoic acid (DHA)	22:6	0.04±0.01[a]	0.05±0.00[a]	-1.24	0.28
Bronsted acid (BSA)	24:2	4.87±1.81[a]	9.45±3.94[a]	-1.06	0.35
Saturated fatty acids (SFAs)	-	22.59±3.06[a]	27.34±4.98[a]	-0.80	0.47
Monounsaturated fatty acids (MUFAs)	-	24.48±2.00[a]	23.59±1.15[a]	0.41	0.70
Polyunsaturated fatty acids (PUFAs)	-	51.92±1.75[a]	48.39±3.87[a]	0.83	0.45
Total fatty acids (TFAs)	-	100.00±0.00	100.00±0.00	-	-
n3:n6	-	757.31±182.14[a]	369.74±205.38[a]	1.41	0.23
EPA:ARA	-	33.44±6.14[a]	32.85±3.86[a]	0.08	0.94
DHA:EPA	-	0.003±0.00[a]	0.003±0.00[a]	-0.62	0.57

Values are presented as mean ± standard error (SE) where n = 3 samples per treatment. Same superscript letters indicate no significant variation in fatty acid content among different dietary treatments ($p < 0.05$).
